# Effect of rearfoot valgus on biomechanics during barbell squatting: A study based on OpenSim musculoskeletal modeling

**DOI:** 10.3389/fnbot.2022.832005

**Published:** 2022-08-09

**Authors:** Zhenghui Lu, Xin Li, Ming Rong, Julien S. Baker, Yaodong Gu

**Affiliations:** ^1^Faculty of Sports Science, Ningbo University, Ningbo, China; ^2^Department of Sport and Physical Education, Centre for Health and Exercise Science Research, Hong Kong Baptist University, Hong Kong, China

**Keywords:** barbell squat, foot pronation, OpenSim, biomechanics, valgus

## Abstract

**Background:**

Barbell squats are commonly used in daily training and rehabilitation. Injuries are not common when the posture is standard, but the wrong posture can lead to injuries. Rearfoot valgus is a common foot abnormality that may increase the risk of injury during sports. The purpose of this study was to compare the biomechanics of lower limbs in normal foot and valgus patients during barbell squat.

**Methods:**

In this study, 10 participants with normal foot shape and 10 participants with rearfoot valgus were enrolled. The joint angle, joint moment, and range of motion of hip, knee, and ankle joints were collected under 0, 30, and 70% one-repetition maximum (RM) load, where discrete data are statistically analyzed using the independent sample *t*-test, and continuous data are statistically analyzed using one-dimensional parameter statistical mapping.

**Results:**

In barbell squats, the range of motion and the joint moment of the hip, knee, and ankle in the rearfoot valgus participants were significantly larger than those in normal foot participants (*p* < 0.05). The participants with rearfoot valgus had a more significant knee valgus angle when squatting to the deepest (*p* < 0.05). In addition, with the increase in load, the participants with rearfoot valgus showed greater standardized medial knee contact force (*p* < 0.05). In the process of barbell squats, the participants with rearfoot valgus showed no significant difference in the foot valgus angle when compared with the normal foot shape (*p* > 0.05).

**Conclusions:**

The valgus population showed a greater range of joint motion when performing barbell squats and showed genu valgus and greater medial knee contact force, which may increase the risk of musculoskeletal and soft tissue damage such as meniscus wear. In addition, there was no significant difference in the rearfoot valgus angle between people with rearfoot valgus and people with normal foot shape during squatting, so barbell squatting may correct valgus to a certain extent.

## Introduction

The barbell squat is a form of weight-bearing, starting from the standing position to lower the hips and then standing up again. The barbell back squat is one of the most frequently used exercises in training and rehabilitation (Schoenfeld, 2010; Whitting et al., 2016; Lee et al., 2019; Lu et al., 2020; Corradi et al., 2021). If performed properly, injuries are uncommon (Dempster et al., 2021), but the wrong technique might lead to serious maladies (Schoenfeld, 2010). Numerous studies have investigated squat-related injuries, and the contact forces at the knee and hip during squat have been widely studied (Schoenfeld, 2010; Bengtsson et al., 2018; Lee et al., 2019). The study of Siewe et al. has shown that 40.8% of powerlifters had experienced lumbar spine injury (Siewe et al., 2011). In addition, according to a review of strength lifting Injuries (Bengtsson et al., 2018), most research and clinical reports about squat damage focus on knee joint injuries. In addition, Sgarlato et al. stressed that during locomotion, abnormal rearfoot valgus might cause postural abnormalities, leading to foot pain and postural symptoms (Sgarlato, 1988). Excessive valgus of the foot may lead to knee valgus (Bell et al., 2008; Li et al., 2021), which may increase the pressure on the anterior ligament and lead to injury (Markolf et al., 1990). However, as far as we know, the squatting of people with the valgus foot has not been thoroughly studied.

Humans will display different postures during the movement, and the change in joint stress will be accompanied (Mei et al., 2015; Li and Gu, 2018; Gu and Sun, 2019). In a study investigating running injuries, James et al. found that valgus at the ankle joint leads to internal rotation of the tibia, which can lead to pain in the knee joint (James et al., 1978). In the study of Levinger and Gilleard, it was also found that patients with patellar joint pain tend to have more valgus feet when standing at rest (Levinger and Gilleard, 2004). In a previous study (Mei et al., 2019), Mei et al. found that prolonged running-induced rearfoot valgus is associated with changes in contact forces on the knee. Valgus foot postures can move the load of the knee joint to the medial side of the leg, which may be related to medial knee arthritis and medial stress syndrome of the knee joint (Boocock et al., 2009). It might be reasonable to assume people with rearfoot valgus may also face the prospect of higher risk of injury during barbell squatting. Therefore, it is essential to analyze the moment and the knee joint's internal and external contact forces in patients with rearfoot valgus and take measures in advance to prevent the risk of sports injury. In addition, too much extra weight is also one of the main factors that increase the risk of injury (Mannis, 1983). When the extra load is raised, the joint load, the ability to control the movement, symmetry, and stability are usually reduced. For patients with rearfoot valgus, it may mean a more severe degree of rearfoot valgus (Waclawski et al., 2015; Xiang et al., 2022), which may have serious consequences (Cote et al., 2005; Zhang and Lu, 2020). However, there are still few studies on the biomechanics of lower limbs during weight-bearing barbell squats in people with rearfoot valgus, and more evidence is needed that the heel valgus angle may affect lower limb joints during squats.

OpenSim is open-source software for visual simulation and analysis of the human musculoskeletal system (Delp et al., 2007), which can adjust the model muscle's start and stop point and insertion point and improve the muscle inaccuracy caused by scaling to a certain extent. This study uses OpenSim muscle and bone modeling technology and a customized squat movement model to compare the biomechanical differences of lower limbs between people with valgus foot and normal foot shape during barbell squatting. Suppose people with valgus have a more significant medial knee joint reaction force during squatting, their risk of injury may be higher, which is of guiding significance for people with the valgus foot to make a training plan.

## Materials and methods

A total of 20 participants were recruited for this study, and each participant performed barbell squats using 0, 30, and 70% RM in a laboratory environment. Joint angles, joint moments, and joint range of motion were calculated for the hip, knee, and ankle joints during exercise using OpenSim, with discrete data statistically analyzed using independent sample *t*-tests and continuous data statistically analyzed using one-dimensional parametric plots. The detailed description of participants' information, experimental design, musculoskeletal model, data processing, model validation methods, and statistical analysis methods are shown.

### Participant

A total of 16 college students were recruited in the study (female/male: 10/10; age: 23.17 ± 1.34 years; weight: 65.67 ± 11.38 kg; height: 1.72 ± 0.07 m). The participants were very healthy, had no history of low back pain or lower limb injury, and had not done any form of exercise in the 48 h before the experiment. In the recruitment stage, the feet of the participants were screened according to the international Foot Posture Index, and the participants with hallux valgus, high arch, and valgus were excluded. On the coronal plane, the angle between the link of heel and ankle joint center and the tibia was larger in the participants with rearfoot valgus when standing upright (valgus angle >5°), and the ankle joint of the normal foot-shaped participants was almost vertical when the foot was erect (<5°). All the participants knew the purpose of the test, method, and steps and signed the informed consent form. The test was approved by the Scientific Research Ethics Committee of Ningbo University.

### Experimental design

**Figure 1:**
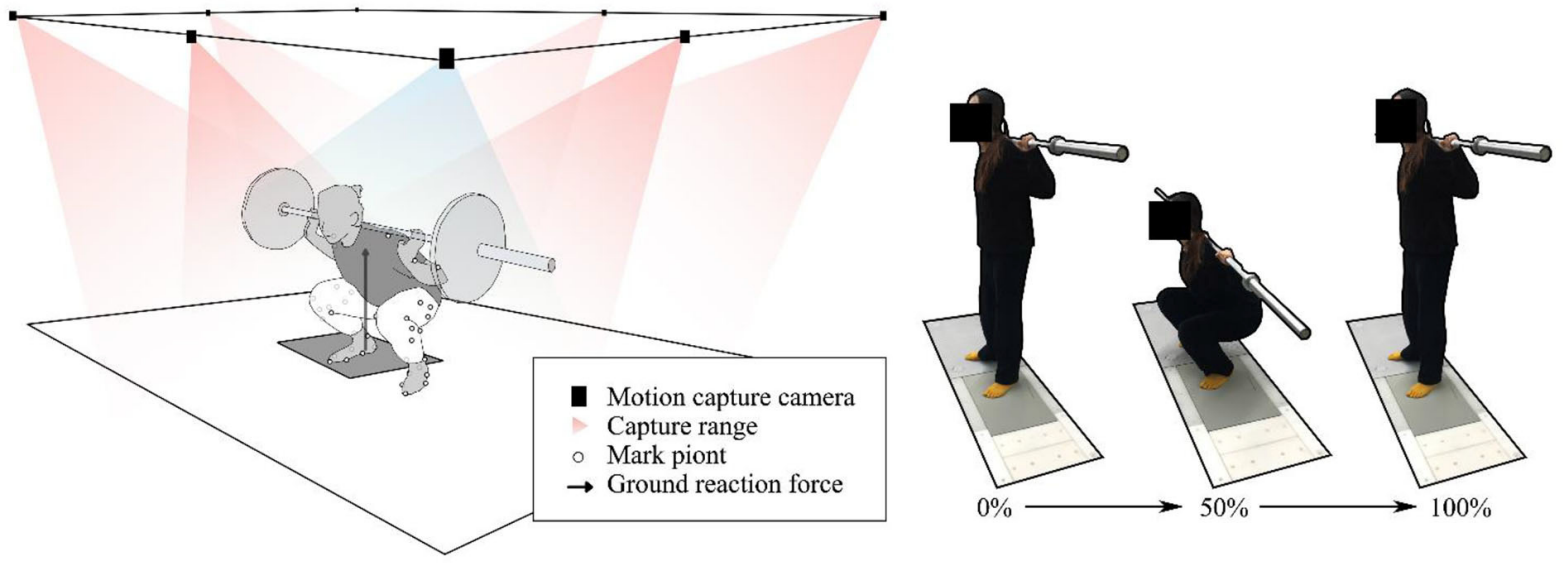
Experimental process, **(left)** participant motion capture setup, and **(right)** squat phase.

A foot scanning machine (VAS-39, Ortho Baltic, LITHUANIA) was used to scan the posture of the participants' feet when standing still. As shown in Figure 1, the Vicon 3D motion capture system (Vicon Metrics Ltd., Oxford, United Kingdom) of eight infrared cameras and the AMTI 3D dynamometer (AMTI, Watertown, Massachusetts, USA) are used to synchronously collect the trajectory of the markers and the data of the ground reaction force. The EMG signals of rectus femoris, biceps femoris muscle, tibialis anterior muscle, and gastrocnemius muscle were collected synchronously with 1,000 Hz frequency by using a wireless Delsys EMG test system (Delsys, Boston, Massachusetts, US) for model verification.

The maximum isometric muscle strength test measures the maximum voluntary contraction (MVC) of muscles. The original EMG signal was first filtered by bandpass fourth-order Butterworth filter in the frequency range of 100–500 Hz in Delsys EMG works Analysis software, and the amplitude analysis was carried out by root mean square (RMS) calculation. The MVC and standardized activity value of each action is output. EMG activity was calculated from 0 (0, completely inactive) to 1 (100%, fully activated) through the test root mean square amplitude/MVC root mean square amplitude.

All the participants received the guidance of the relevant practitioners, carried out the squat ultimate weight test, and carried out 5 min of warm-up and squat exercises before the experiment. In the experiment, the participants adopted a high-level position, placed the right foot stepped into the force measuring platform, began to record the movements in the upright position of the knee joint to ensure that the pelvis squatted to the deepest place, and then extended the hip, knee, and ankle joint to restore the upright state. During the squat, the participants were asked to complete the movement as evenly as possible and not to use burst squats or bounce off the bottom. Each participant used the weight of no weight, 30% 1RM, and 70% 1RM to collect three successful squatting data, with a squatting interval of 4 min each to ensure full rest.

### Musculoskeletal model, data processing, and model verification

In this study, a modified model customized for squatting was used. Based on adjusting the range of motion of the knee joint (Lu et al., 2020), the range of motion of the subtalar joint was opened to obtain the external and internal rotation angles of the foot and the degrees of freedom on the rotation and coronal plane of the knee joint. To obtain the moment and adduction moment of the knee joint, the original squat model (Catelli et al., 2019) adjusted the wrapping surface of the hip and knee muscles based on the whole body model (Delp et al., 2007; McNamara and Stearne, 2010) to allow a higher range of motion.

**Figure 2:**
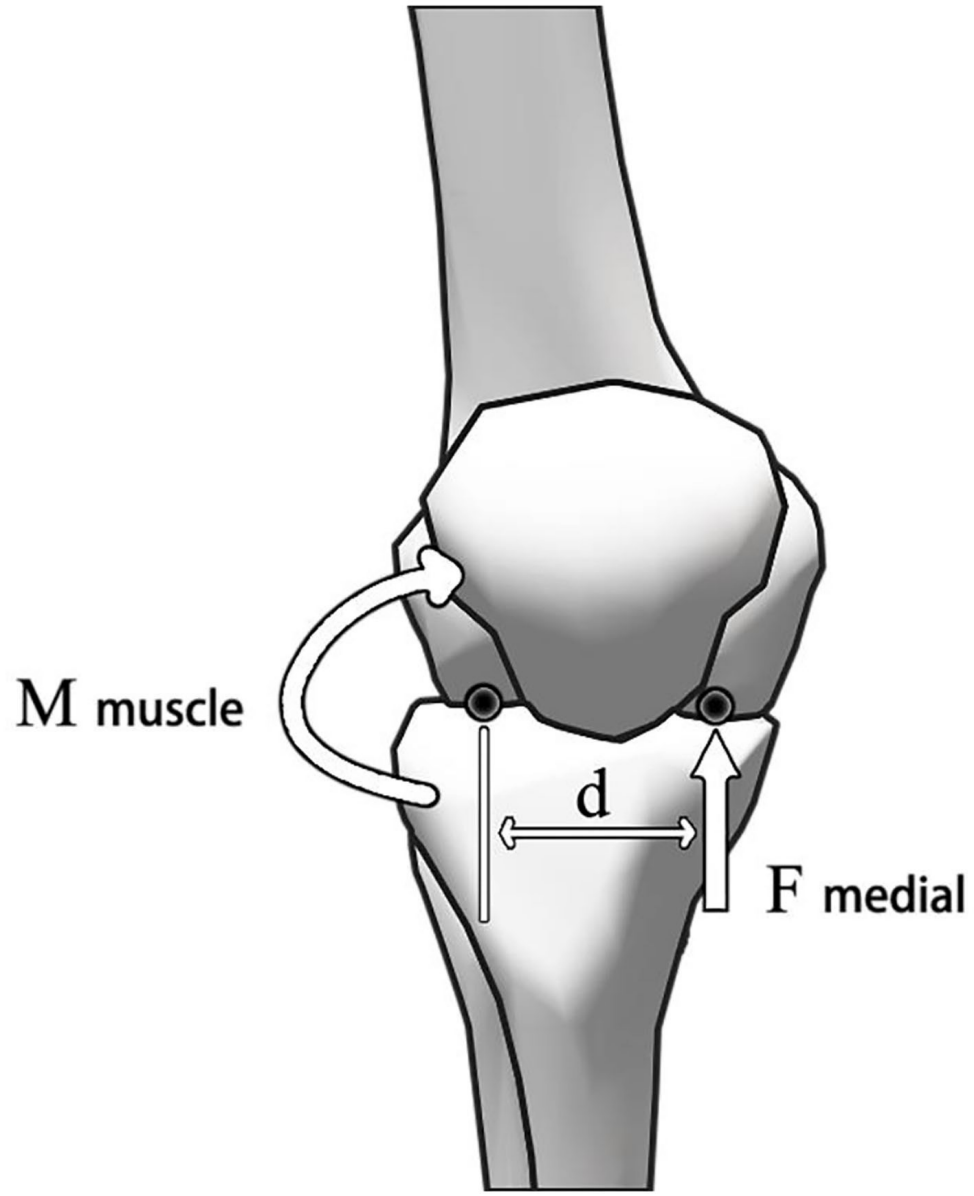
Calculation of medial contact force of knee joint.

The trajectory of the marked points and the ground reaction data collected by the experiment were processed and converted by the self-designed MATLAB program, and the OpenSim workflow was carried out according to the published scheme (Delp et al., 2007). First of all, the weight of the marked points in the model was manually adjusted. The model was scaled to meet the anthropometric characteristics of the participants so that the root mean square error between the marked points and the virtual marked points in the experiment was <0.02 meters. The maximum error was <0.04 m. Second, the inverse kinematics algorithm was used to calculate the joint angle of the minimum error between the marked point and the virtual marked point in the experiment. Then, the inverse dynamics algorithm was used to calculate the joint moment. Then, the static optimization algorithm was run, which uses the minimized sum of squares of muscle activation to calculate the degree of muscle activation and muscle strength (Delp et al., 2007; DeMers et al., 2014; Lerner et al., 2014; Mei et al., 2019). Finally, the analysis tool was used to calculate the total contact force of the knee joint. The medial contact force of the knee joint was determined by the static balance of the contact point (Winby et al., 2009; Kumar et al., 2012). According to the method described by Schipplein and Andriacchi, the proportion of the contact force passing through the medial space of the tibiofemoral joint was estimated. Using this method, the position of the acting point of the medial and lateral contact forces was determined to be 25% of the width of the tibia from the center of the knee joint (Schipplein and Andriacchi, 1991). As shown in Figure 2, referring to the method described by Pauline in their study (Gerus et al., 2013), the abduction moment of the knee joint is output through the inverse kinetics in OpenSim, which is balanced by the muscle moment relative to the outer contact point (i.e., the product of the muscle force and the moment arm calculated by the OpenSim static optimization algorithm) and the unknown inside contact force. This process was repeated in each time frame. In addition, the joint moment and joint contact force obtained were normalized by dividing the sum of bodyweight and barbell.

In addition to recording muscle activity during squatting, the level of muscle activity during MVC was collected before the test. The EMG data were processed using the root mean square (RMS) algorithm in Delsys EMG works analysis software. The standardized muscle activation level was obtained by dividing the muscle activity level during squatting by MVC ('0' for complete inactivation and '1' for full activation). The muscle activation level measured by the experiment was compared with the muscle activation obtained by the static optimization algorithm to complete the model verification.

### Statistical analysis

As shown in Figure 1, in this study, squatting was divided into a. descent stage, from the body upright knee flexion to squatting to the deepest position (0%-50%), and b. ascending phase, from the squatting to deepest position to upright position (51%-100%). An independent sample *t*-test was used to analyze the range of motion and peak moment of the normal foot and rearfoot valgus participants during squatting under different loads. It includes flexion (+)/extension (-), abduction (-)/adduction (+), ankle dorsiflexion (+)/plantarflexion (-), and supination (+)/valgus (-) of hip and knee joints. Because the joint angle, joint moment, and joint reaction force have one-dimensional time-varying characteristics, MATLAB is used to run the open-source one-dimensional parameter statistical mapping program for the independent sample *t*-test, and the significance level is set at 0.05.

## Results

### Model verification

**Figure 3:**
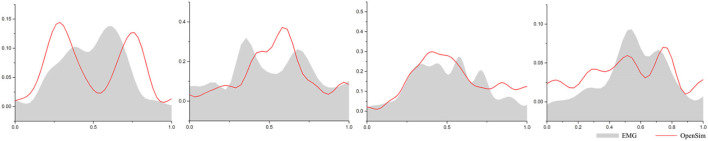
Comparison of rectus femoris, gastrocnemius, biceps femoris, and tibialis anterior muscle activation level obtained by EMG signal and OpenSim optimization algorithm.

As shown in Figure 3, the muscle activation calculated by OpenSim static optimization tool during barbell squatting is similar to the EMG signal activity recorded in the experiment, indicating that the data of the OpenSim model in this study is reliable (Hamner and Delp, 2013; Rajagopal et al., 2016). The joint angle and joint moment obtained by OpenSim inverse kinematics and inverse kinetics algorithm are similar to those in previous studies (Lu et al., 2020).

### Kinematics

The maximum joint angle of participants squatting under three different load conditions is shown in Table 1. Without additional weight load, the hip joint's maximum flexion, and abduction angle, the maximum flexion angle of the knee joint, and the maximum dorsiflexion angle of the ankle joint in the rearfoot valgus group were significantly larger than those in the normal foot group (*p* < 0.05). Under the condition of 30% 1RM load, the maximum flexion angle of the hip joint and the maximum flexion angle of the knee joint and ankle joint of people with valgus foot were significantly higher than those with a normal foot shape (*p* < 0.05). Under the condition of 70% 1RM load, the maximum flexion and abduction angle of the hip joint, the maximum flexion angle and adduction of knee joint, and the maximum dorsiflexion angle of the ankle joint in rearfoot valgus participants were significantly higher than those in normal foot participants (*p* < 0.05).

**Table 1:** Peak joint angle.

**Index**	**Rearfoot valgus group**			**Control group**		
	**0%**	**30%**	**70%**	**0%**	**30%**	**70%**
Hip flexion (°)	99.49 ± 11.43^*^	103.12 ± 11.73^*^	103.78 ± 9.69^*^	91.97 ± 5.16	92.63 ± 8.10	92.87 ± 11.04
Hip abduction (°)	20.23 ± 3.75^*^	23.74 ± 6.13	23.79 ± 5.91	25.60 ± 2.50	26.12 ± 3.81	24.36 ± 4.44
Knee flexion (°)	143.67 ± 6.59^*^	145.12 ± 4.60^*^	144.66 ± 7.59^*^	134.06 ± 5.84	132.21 ± 4.57	131.21 ± 5.58
Knee adduction (°)	6.25 ± 5.82	7.62 ± 6.65	8.86 ± 7.32^*^	6.70 ± 2.78	4.63 ± 4.60	2.70 ± 5.11
Knee abduction (°)	5.90 ± 3.51	5.92 ± 3.50	6.46 ± 3.32	4.21 ± 3.19	5.21 ± 3.50	7.39 ± 4.16
Ankle dorsiflexion (°)	37.81 ± 1.43^*^	38.24 ± 1.26^*^	37.31 ± 3.12^*^	33.97 ± 3.18	34.72 ± 4.28	33.85 ± 5.07
Foot valgus (°)	17.11 ± 6.21	18.01 ± 5.26	16.75 ± 2.59	21.04 ± 5.46	15.70 ± 7.83	15.13 ± 7.28

* *Significant differences with the control group*.

The ROM data of squatting under three different load conditions are shown in Table 2. Without additional weight loading, the range of motion of the hip joint in the valgus foot was smaller than that in the normal foot shape (*p* < 0.05), and the range of motion of the knee joint and ankle joint in the sagittal plane was larger (*p* < 0.05). Under 30% and 70% 1RM load, the range of flexion and extension of hip joint, knee joint, and ankle joint of the participants with rearfoot valgus was larger (*p* < 0.05), and the knee joint showed a larger range of motion on the coronal plane (*p* < 0.05). In addition, the knee internal rotation moment of the participants with rearfoot valgus was significantly greater than that of the participants with normal feet (*p* < 0.05).

**Table 2:** Range of motion of the joint.

**Index**		**Rearfoot valgus group**			**Control group**		
	**0%**	**30%**	**70%**	**0%**	**30%**	**70%**
Hip (°)	Sagittal	87.21 ± 7.82	87.56 ± 8.22^*^	88.01 ± 5.25^*^	85.47 ± 6.93	79.42 ± 5.68	79.04 ± 8.96
	Coronal	14.34 ± 2.38^*^	17.99 ± 3.36	17.62 ± 3.63	16.53 ± 2.25	16.83 ± 2.07	15.24 ± 2.58
	Cross section	24.93 ± 10.11	29.89 ± 11.48	31.86 ± 11.68	24.04 ± 6.98	23.95 ± 10.76	24.08 ± 8.93
Knee (°)	Sagittal	139.34 ± 5.86^*^	141.18 ± 4.10^*^	140.57 ± 5.72^*^	127.56 ± 9.06	125.89 ± 7.74	123.57 ± 8.66
	Coronal	12.15 ± 3.55	13.54 ± 4.42^*^	15.32 ± 4.72^*^	11.06 ± 2.25	9.49 ± 2.85	10.10 ± 2.51
	Cross section	35.47 ± 5.98	39.16 ± 7.51^*^	38.05 ± 6.20	35.22 ± 5.35	31.15 ± 9.93	31.92 ± 9.21
Ankle (°)	Sagittal	41.68 ± 2.49^*^	45.11 ± 3.49^*^	44.91 ± 2.61^*^	37.35 ± 2.48	38.97 ± 2.66	37.64 ± 3.50
	Valgus/Supination	14.88 ± 6.06	17.37 ± 6.39	17.62 ± 7.52	18.49 ± 4.11	14.87 ± 4.20	14.43 ± 3.60

* *Significant differences with the control group*.

**Figure 4:**
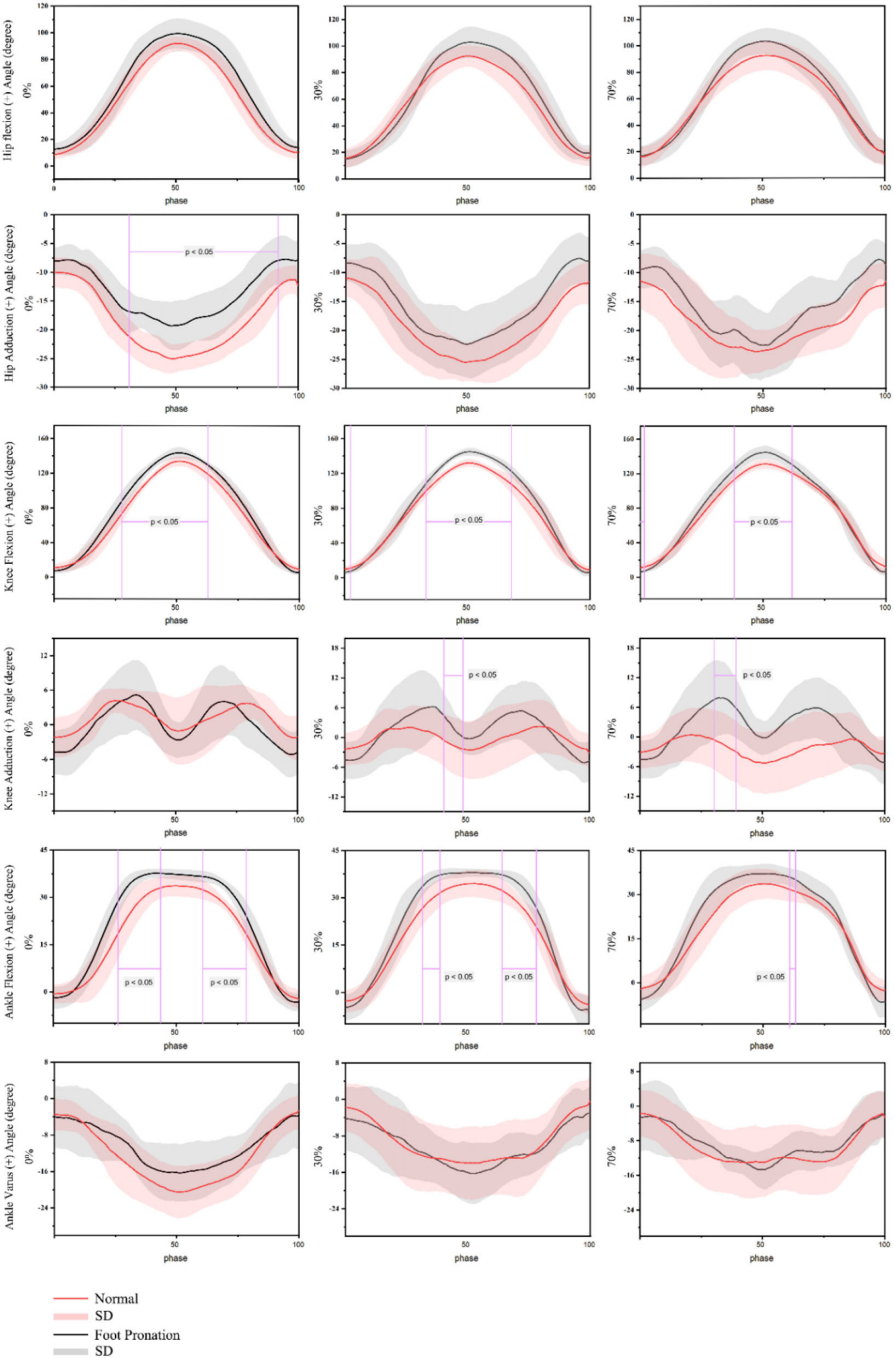
Angle of barbell squat joint in people with pronation foot and those with normal foot shape.

The data of the change of joint angle overtime under three different load conditions are shown in Figure 4. The hip abduction angle of normal foot participants was significantly larger than that of valgus foot participants, with the range of 31–93% of squatting without weight-bearing (*p* < 0.05). The flexion angle of the knee joint of rearfoot valgus participants during squatting was significantly larger than that of normal foot participants (*p* < 0.001), and the differences occurred near 50% of the whole squatting cycle when squatting to the deepest without additional load (28%-63%), weight-bearing 30% 1RM (0–2%; 33–67%), and weight-bearing 70% 1RM (0–2%; 38–62%). The knee joints of the two groups of participants showed adduction during squatting and lifting. The knee adduction angle of the rearfoot valgus participants was larger than that of the normal foot-shaped participants. There was a significant difference in 41–49% of 30% 1RM load, the knee adduction angle of the participants with heel valgus was significantly greater at 70%1 RM load during 31–39% of the squat (*p* < 0.05). The ankle dorsiflexion angle of the participants with the valgus foot was also significantly higher than that of the participants with a normal foot shape (*p* < 0.05) at no external load (26–44%; 61–79%), 30% 1RM load (32–39%; 64–78%), and 70% 1RM load (61–64%).

### Joint moment

The peak joint moment of participants squatting under three different load conditions is shown in Table 3. Under the condition of no additional weight load, the peak abduction moment of the hip joint, the peak adduction moment of the knee joint, and the peak plantarflexion and valgus moment of the ankle joint in the rearfoot valgus group were significantly higher than those in the normal foot group (*p* < 0.05). The peak extension moment of the knee joint was significantly lower than that in the normal foot group (*p* < 0.05). Under the condition of 30% 1RM load, the peak extension moment of the hip joint, the peak abduction moment of the hip joint, the peak adduction moment of the knee joint, the peak extension moment of the ankle joint, and the internal rotation moment of the foot were significantly higher than those of a normal foot shape (*p* < 0.05), and the peak extension moment of the knee joint was significantly lower than that of a normal foot shape (*p* < 0.05). Under the condition of 70% 1RM load, the peak extension moment of hip joint, the peak abduction moment of the hip joint, the peak external moment of knee joint, the peak adduction moment of knee joint, the peak plantarflexion moment of the ankle joint, and the internal rotation moment of the foot were significantly higher than those of a normal foot shape (*p* < 0.05), and the peak extension moment of the knee joint was significantly lower than that of a normal foot shape (*p* < 0.05).

**Table 3:** Peak joint moment.

**Index**	**Rearfoot Valgus Group**			**Control Group**		
	**0%**	**30%**	**70%**	**0%**	**30%**	**70%**
Hip flexion moment (Nm/kg)	−0.54 ± 0.18	−0.78 ± 0.23^*^	−0.95 ± 0.16^*^	−0.45 ± 0.04	−0.61 ± 0.12	−0.74 ± 0.09
Hip adducton moment (Nm/kg)	−0.25 ± 0.05^*^	−0.29 ± 0.03^*^	−0.35 ± 0.04^*^	−0.16 ± 0.03	−0.17 ± 0.06	−0.18 ± 0.05
Hip internal rotation moment (Nm/kg)	−0.40 ± 0.16	−0.34 ± 0.11	−0.32 ± 0.10	−0.38 ± 0.08	−0.38 ± 0.06	−0.36 ± 0.04
Knee flexion moment (Nm/kg)	−1.05 ± 0.17^*^	−1.02 ± 0.17^*^	−0.92 ± 0.17^*^	−1.24 ± 0.13	−1.21 ± 0.20	−1.10 ± 0.15
Knee internal rotation moment (Nm/kg)	−0.15 ± 0.10	−0.14 ± 0.10	−0.15 ± 0.10^*^	−0.12 ± 0.05	−0.09 ± 0.04	−0.07 ± 0.04
Knee adduction moment (Nm/kg)	0.35 ± 0.11^*^	0.27 ± 0.08^*^	0.28 ± 0.07^*^	0.23 ± 0.06	0.21 ± 0.04	0.20 ± 0.05
Ankle extension moment (Nm/kg)	0.53 ± 0.10^*^	0.62 ± 0.11^*^	0.64 ± 0.09^*^	0.37 ± 0.16	0.39 ± 0.19	0.49 ± 0.19
Foot valgus moment (Nm/kg)	0.20 ± 0.03^*^	0.24 ± 0.04^*^	0.26 ± 0.05^*^	0.16 ± 0.05	0.16 ± 0.06	0.18 ± 0.08

* *Significant differences with the control group*.

**Figure 5:**
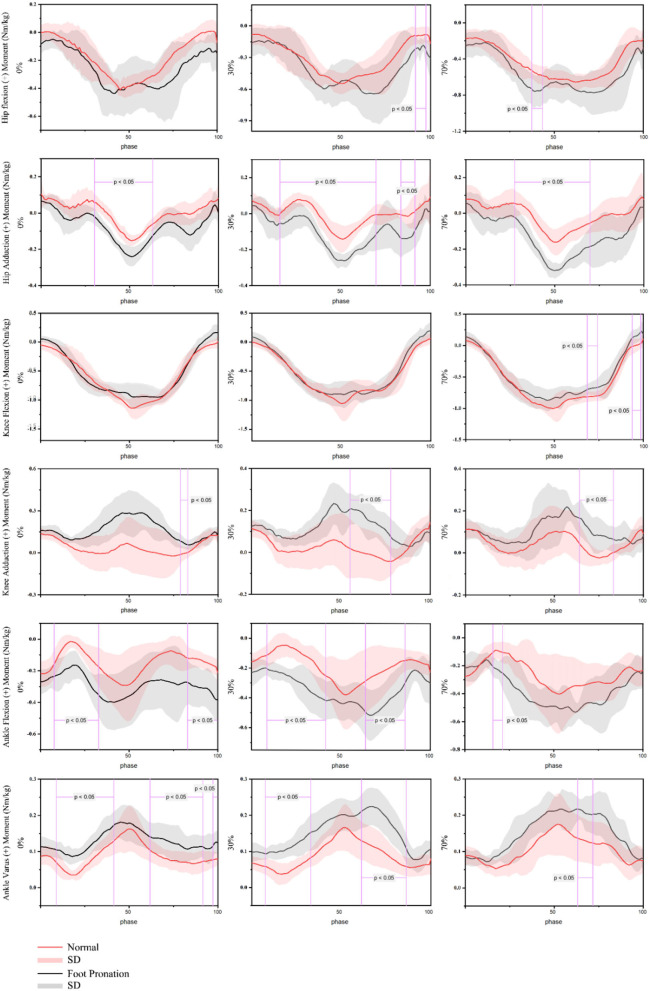
Joint moment of barbell squatting in people with pronation foot and normal foot shape.

The data of the variation of the joint moment with time under three different load conditions are given in Figure 5. In the case of 30% 1RM (91–98%) and 70% 1RM (37–43%) load, the hip joint extension moment of rearfoot valgus participants was significantly larger than that of normal foot participants (*p* < 0.05). There was no significant difference when there was no extra weight load. In addition, it is worth noting that the participants of rearfoot valgus appeared two peaks of hip extension moment in the process of squatting, which was before and after squatting to the deepest position, while in normal foot participants, there was only one wave peak of hip extension moment in the whole exercise cycle, which appeared in the deepest part of squatting in the case of no weight and 30% 1RM, and the lifting phase in the case of 70% 1RM. The hip abduction moment of participants with rearfoot valgus was significantly higher than that of participants with a normal foot shape under the conditions of no weight-bearing (30–63%), 30% 1RM load (17–71%; 84–92%), and 70% 1RM load (28–70%). The hip abduction moment of participants with rearfoot valgus was significantly larger than that of participants with a normal foot shape. The knee extension moment of participants with rearfoot valgus was significantly lower than that of participants with a normal foot at 70% 1 RM load (68–74%; 93–99%) (*p* < 0.05). The knee adduction moment of the participants with rearfoot valgus also had two peaks on both sides of the action cycle, and it was significantly larger than that of normal foot participants both under no weight-bearing (79–83%), 30% 1RM (55–78%), and 70% 1RM (64–83%) loads (*p* < 0.05). The ankle extension moment and ankle adduction moment of rearfoot valgus participants were significantly larger than those of normal foot participants at the initial stage of squatting and at the end of lifting (*p* < 0.05).

### Knee joint reaction force

The peak joint moment of participants squatting under three different load conditions is shown in Table 4. Under the condition of no additional weight load and 30% 1RM load, there was no significant difference in the total knee contact force between the rearfoot valgus patients and the normal foot shape group (*p* > 0.05). Still, the medial contact force of the knee joint was significantly higher than that of the normal foot shape group (*p* < 0.05). In the case of 70% 1RM load, the peak total knee contact force and peak medial knee contact force of people with valgus feet were significantly higher than those with a normal foot shape (*p* < 0.05).

**Table 4:** Peak knee joint reaction force.

**Index**	**Rearfoot Valgus Group**			**Control Group**		
	**0%**	**30%**	**70%**	**0%**	**30%**	**70%**
Total contact force of knee (N/kg)	24.09 ± 2.65	22.76 ± 5.69	21.78 ± 4.06^*^	23.26 ± 6.03	19.58 ± 3.62	18.38 ± 4.13
Medial contact force of knee (N/kg)	5.66 ± 2.80^*^	3.95 ± 1.54^*^	4.23 ± 0.82^*^	2.38 ± 1.63	1.81 ± 1.20	1.78 ± 1.08

* *Significant differences with the control group*.

**Figure 6:**
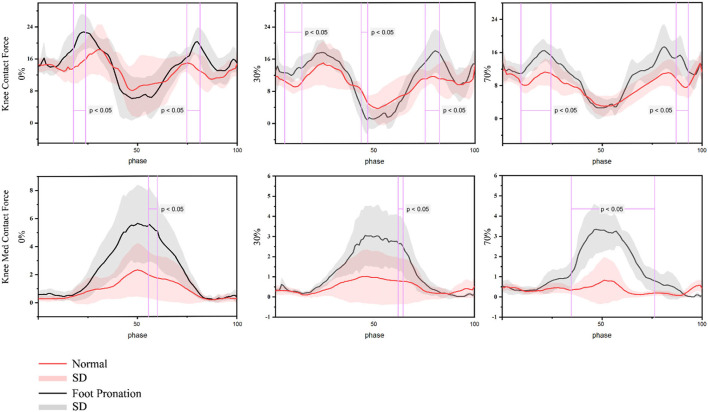
Contact force of the knee joint of the barbell squat in people with pronation foot and normal foot shape.

The change data of joint moment with time under three different load conditions are given in Figure 6. The total knee contact force of rearfoot valgus participants and normal foot participants was similar. Under no extra load (18–24%; 75–82%), 30% 1RM load (5–13%; 43–46%; 75–83%), and 70% 1RM load (9–24%; 87–93%), the total knee contact force of the participants with rearfoot valgus was significantly greater than that of the participants with normal foot during the initial descending and ascending phases. The medial contact force of the knee joint in the participants with rearfoot valgus was greater than that in the participants with normal foot. When there was no extra load (56–60%) and 30% 1RM load (61–64%), the difference was mainly in the lifting phase (*p* < 0.05). When using 70% 1RM load, the medial contact force of the knee joint was significantly greater during 35–76% of the squat phase (*p* < 0.05).

## Discussion

Barbell squatting is a common exercise used to strengthen lower limbs and drive away strength in training and rehabilitation (Chandler and Stone, 1991; Hickson et al., 1994; Thein and Brody, 1998; Gullett et al., 2009; Sato et al., 2012; Sinclair et al., 2015), and pain is considered to be a common problem in squat training. This kind of pain may be related to excessive load, large-scale exercise, and errors in motor skills (Bengtsson et al., 2018). The foot is the beginning of contact with the ground during exercise, and there is evidence that there is a link between excessive rearfoot valgus and knee valgus (Joseph et al., 2008), which is an action error that may lead to knee joint injury (Hetsroni et al., 2006), and overload may cause additional risk. However, at present, there are only few studies on the biomechanics of lower limbs during barbell squatting in people with valgus feet, and more evidence is needed. Therefore, in this study, the customized OpenSim muscle-bone model was used to compare the joint angle, joint moment, and knee contact force of different types of weight-bearing barbell squatting between the rearfoot valgus group and the normal foot shape group.

The study found that people with valgus feet showed a larger flexion angle of hip, knee, and ankle during barbell squatting, which was partly consistent with the study of Lee et al. (2015). In line with the results of Jong et al., people with valgus feet have a larger dorsal flexion angle of the ankle during squatting, which may be due to the increased flexibility of the ankle joint (Gu et al., 2014). However, in the results of Jong et al., the flexion angle of the hip joint of the rearfoot valgus group was significantly smaller when squatting. By contrast, in this study, the range of motion of the hip and knee joint in the sagittal plane of the rearfoot valgus participants was significantly greater during the barbell squat, which may be due to the difference in movement patterns. In Jong et al.'s study, squatting lasted only to 90° knee flexion, but in this study, the participants were asked to squat as deep as possible, a difference that may have led to differences in results. In Jong et al.'s study, participants only squatted in one direction, while in this study, the participants dropped first and then rose. People with valgus feet may be more likely to increase their range of motion and use muscle stretch reflexes to complete deep squats. The study also observed that people with rearfoot valgus has a smaller abduction angle of the hip joint and the larger abduction angle of the knee joint when squatting to the deepest position, which means that knee valgus may occur, which is a common movement error and may increase the risk of knee joint injury during exercise (Markolf et al., 1995; Rodrigues et al., 2020). In addition, it is worth noting that in a study investigating the effects of rearfoot valgus on adolescent walking kinetics (Caravaggi et al., 2018), participants with valgus feet had more ankle abduction during walking than participants with normal foot shape. This is contrary to the results of this study. In this study on squatting, the participants with valgus feet did not show a larger valgus angle during barbell squatting, which may be because, in the squat movement mode, the distance between the feet was larger than that when standing normally. The feet showed a state of natural adduction, resulting in no significant difference between the angle of the ankle on the coronal plane and the normal foot shape, which is an interesting finding. Maybe it can provide some evidence for squatting to improve rearfoot valgus.

Kinetics results showed that the peak moment of hip extension, hip abduction, knee external rotation, knee adduction, ankle extension, and foot valgus were significantly higher in participants with valgus feet than in participants with normal feet, and knee joint extension moment was significantly lower in participants with valgus feet than that in those normal feet. The difference of joint moment mainly appeared in the descending and rising stages of squatting. This is similar to the results of a previous study by Mei et al. on the effect of rearfoot valgus on a load of lower limbs during running (Mei et al., 2019). Mei et al. found that the participants showed increased static rearfoot valgus after 5 km running on the treadmill, which may increase lower limb joint load. However, the movement pattern of running is different from that of barbell squatting. At present, the research on rearfoot valgus on lower limb biomechanics during barbell squat is very few, and more evidence is needed in future. In addition, it is worth noting that in the process of squatting, there are two peaks in the extension moment of the hip joint and knee joint in the rearfoot valgus group, but only one in the normal foot participants. This may be a mechanism to reduce the load. This mechanism is thought to reduce the load on the joint during exercise (Clarke et al., 1983; Pratt, 1989; Xu et al., 2022), which is also observed in the results of knee joint load. The results showed that when squatting to the deepest position, the total knee contact force of the valgus participants was smaller than that of the normal foot participants, and there was a statistically significant difference in the weight-bearing of 30% 1RM. In addition, the results showed that the contact force of the lateral knee joint increased with the increase in the knee flexion angle during squatting and reached the peak when squatting to the deepest point. The medial contact force of the knee joint of valgus participants was larger than that of normal foot participants, and there was a statistically significant difference between 30% 1RM and 70% 1RM. This is similar to the results of Mei et al. (2019), who found that rearfoot valgus after medium-distance running can increase the medial contact force of the knee joint. This may be because when the rearfoot valgus occurs, both the tibia and femur will rotate internally, the knee joint will appear adduction, and the COP of the knee joint will be offset, resulting in a redistribution of pressure. At present, the research on rearfoot valgus is mainly focused on kinematic analysis to evaluate the effect of rearfoot valgus on barbell squatting. In this study, the OpenSim muscle-bone modeling technique was used to analyze the impacts of rearfoot valgus on the joint angle, joint moment, and knee joint contact force during barbell squatting. The results show that rearfoot valgus can increase the medial contact force of barbell squatting, especially under a high weight load. There is an additional risk of injury. However, this study also has limitations.

The participants in this study were all mild valgus with a valgus foot angle of no more than 30°. The possible effects of severe valgus were not known. In addition, the results of this study can only be inferred that the valgus foot will increase the medial knee pressure. The exact linear relationship between the eversion angle and the pressure is unknown. Therefore, in future research, the sample size should be further expanded to recruit participants with different degrees of rearfoot valgus and quantify the linear relationship between valgus angle and knee joint pressure.

## Conclusion

In this study, the lower limb joint angle, joint moment, and knee contact force of people with different weight-bearing barbell squatting techniques were compared between rearfoot valgus and normal foot people. The results show that (1) the participants of rearfoot valgus have a larger range of motion of lower limb joints in the sagittal plane during barbell squatting, indicating that rearfoot valgus will increase the flexion of the hip, knee, and ankle joint in the process of squatting; (2) the degree of abduction of the hip joint of the participants of rearfoot valgus is lower, indicating that the valgus of the foot during squatting may makes the knee joint more adducted and cause knee valgus, which may lead to knee joint injury; (3) there is no significant difference in the rearfoot valgus angle between rearfoot valgus people and normal foot people during squatting, the distance between participants' feet during squatting is farther than that when standing usually, and the feet are in a state of natural adduction during squatting, so squatting may be a means to correct rearfoot valgus; (4) there is almost no difference in the total contact force of the knee joint between the rearfoot valgus group and the normal foot shape group. Still, the medial contact force of the knee joint of the rearfoot valgus group is significantly higher than that of the normal foot shape crowd. This suggests that foot pronation may lead to a redistribution of knee pressure during deep squats, leading to a higher risk of injury.

## Data availability statement

The raw data supporting the conclusions of this article will be made available by the authors, without undue reservation.

## Ethics statement

The studies involving human participants were reviewed and approved by Ethics Committee of Ningbo University. The patients/participants provided their written informed consent to participate in this study.

## Author contributions

ZL, XL, and YG: conceptualization and software. XL, MR, and JB: methodology and validation. YG and JB: investigation. ZL: writing—original draft preparation. MR, JB, and YG: writing—review and editing. All authors have read and agreed to the published version of the manuscript.

## Funding

This study was sponsored by the National Natural Science Foundation of China (No. 81772423), Key Project of the National Social Science Foundation of China (19ZDA352), Key R&D Program of Zhejiang Province China (2021C03130), Ningbo Public Welfare Science and Technology Plan Project (No. 2019C50095), Health Youth Technical Talent Cultivation Special Fund Project (2020SWSQNGG01), Ningbo Medical Science and Technology Plan (2020Y14), Young Cultivation Fund Project of The Affiliated of School of Medicine of Ningbo University (FYQM-KY-202003), Open Fund Project of Institute of Human Biomechanics of Ningbo University (CJ-HBIO202112), and K.C. Wong Magna Fund in Ningbo University.

## Conflict of interest

The authors declare that the research was conducted in the absence of any commercial or financial relationships that could be construed as a potential conflict of interest.

## Publisher's note

All claims expressed in this article are solely those of the authors and do not necessarily represent those of their affiliated organizations, or those of the publisher, the editors and the reviewers. Any product that may be evaluated in this article, or claim that may be made by its manufacturer, is not guaranteed or endorsed by the publisher.
